# Supplementary material for: Arylamine Analogs of Methylene Blue: Substituent Effect on Aggregation Behavior and DNA Binding
Source: Int J Mol Sci. 2021 May 29;22(11):5847. doi: 10.3390/ijms22115847 (PMC8198855; doi:10.3390/ijms22115847)
Supplement: Supplementary file 1 [file ijms-22-05847-s001.zip › ijms-1237331-supplementary.pdf]

Supplementary information

Arylamine Analogs of Methylene Blue: Substituent Effect on Aggregation Behavior and DNA Binding

Alena Khadieva, Olga Mostovaya, Pavel Padnya, Valeriy Kalinin, Denis Grishaev, Dmitrii Tumakov and Ivan Stoikov

Content

2 .... FIGURE S1. ^{1}H NMR SPECTRUM OF THE COMPOUND PTZA, DMSO-*D*$_6$, 300 K, 400 MHZ.

2 .... FIGURE S2. ^{1}H NMR SPECTRUM OF THE COMPOUND PTZ1, DMSO-*D*$_6$, 300 K, 400 MHZ.

3 .... FIGURE S3. ^{1}H NMR SPECTRUM OF THE COMPOUND PTZ2, DMSO-*D*$_6$, 300 K, 400 MHZ.

3 .... FIGURE S4. ^{13}C NMR SPECTRUM OF THE COMPOUND PTZA, DMSO-*D*$_6$, 300 K, 100 MHZ.

4 .... FIGURE S5. ^{13}C NMR SPECTRUM OF THE COMPOUND PTZ1, DMSO-*D*$_6$, 300 K, 100 MHZ.

4 .... FIGURE S6. ^{13}C NMR SPECTRUM OF THE COMPOUND PTZ2, DMSO-*D*$_6$, 300 K, 100 MHZ.

5 .... FIGURE S7. FT-IR SPECTRUM OF THE COMPOUND PTZA.

5 .... FIGURE S8. FT-IR SPECTRUM OF THE COMPOUND PTZ1.

5 .... FIGURE S9. FT-IR SPECTRUM OF THE COMPOUND PTZ2.

6 .... FIGURE S10. HRMS SPECTRUM OF THE COMPOUND PTZA.

6 .... FIGURE S11. HRMS SPECTRUM OF THE COMPOUND PTZ1.

7 .... FIGURE S12. HRMS SPECTRUM OF THE COMPOUND PTZ2.

7 .... FIGURE S13. 1/[DNA] VERSUS $A_0/(A- A_0)$ PLOT FOR MB-DNA UV-VIS TITRATION.

8 .... FIGURE S14. 1/[DNA] VERSUS $A_0/(A- A_0)$ PLOT FOR PTZ1-DNA UV-VIS TITRATION.

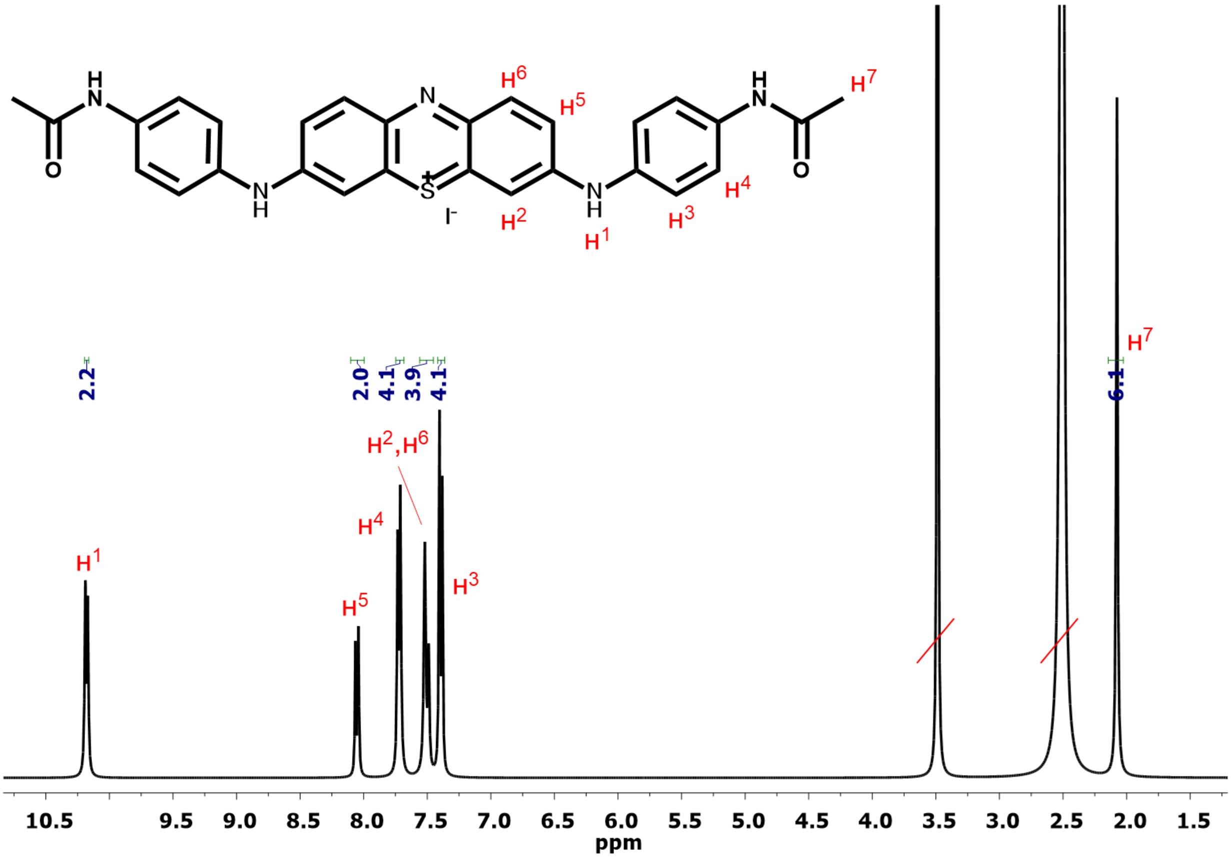

Figure S1. ^{1}H NMR spectrum of the compound **PTZa**, DMSO-d_6, 300 K, 400 MHz.

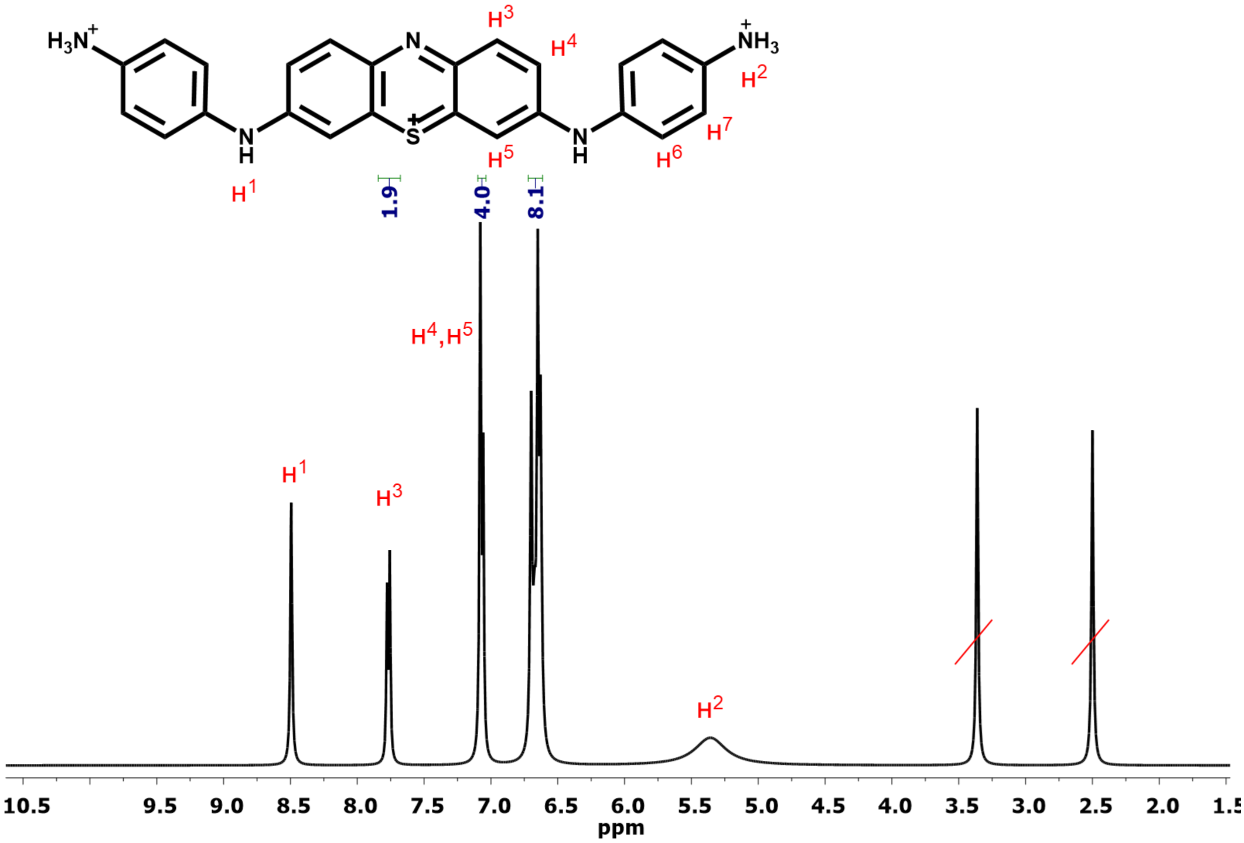

Figure S2. ^{1}H NMR spectrum of the compound **PTZ1**, DMSO-d_6, 300 K, 400 MHz.

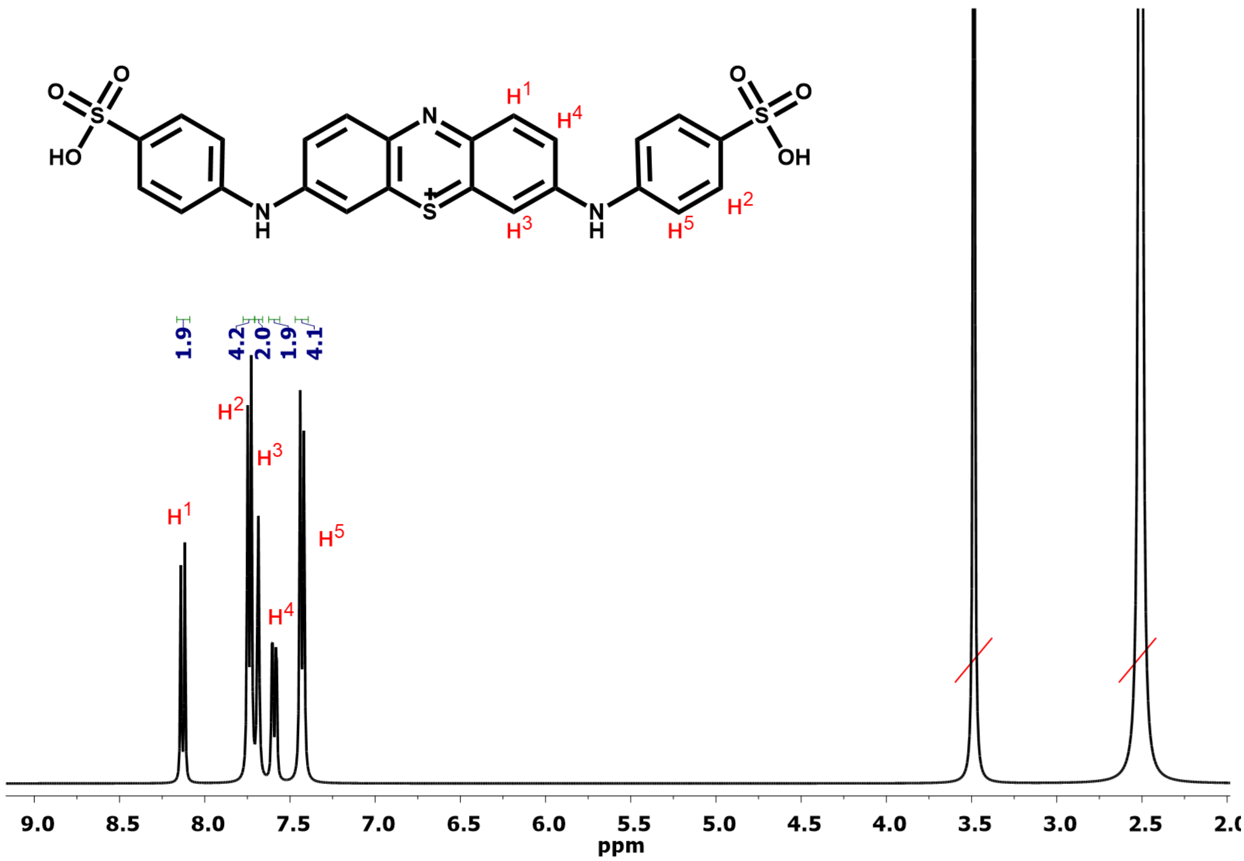

Figure S3. ^{1}H NMR spectrum of the compound **PTZ2**, DMSO-d_6, 300 K, 400 MHz.

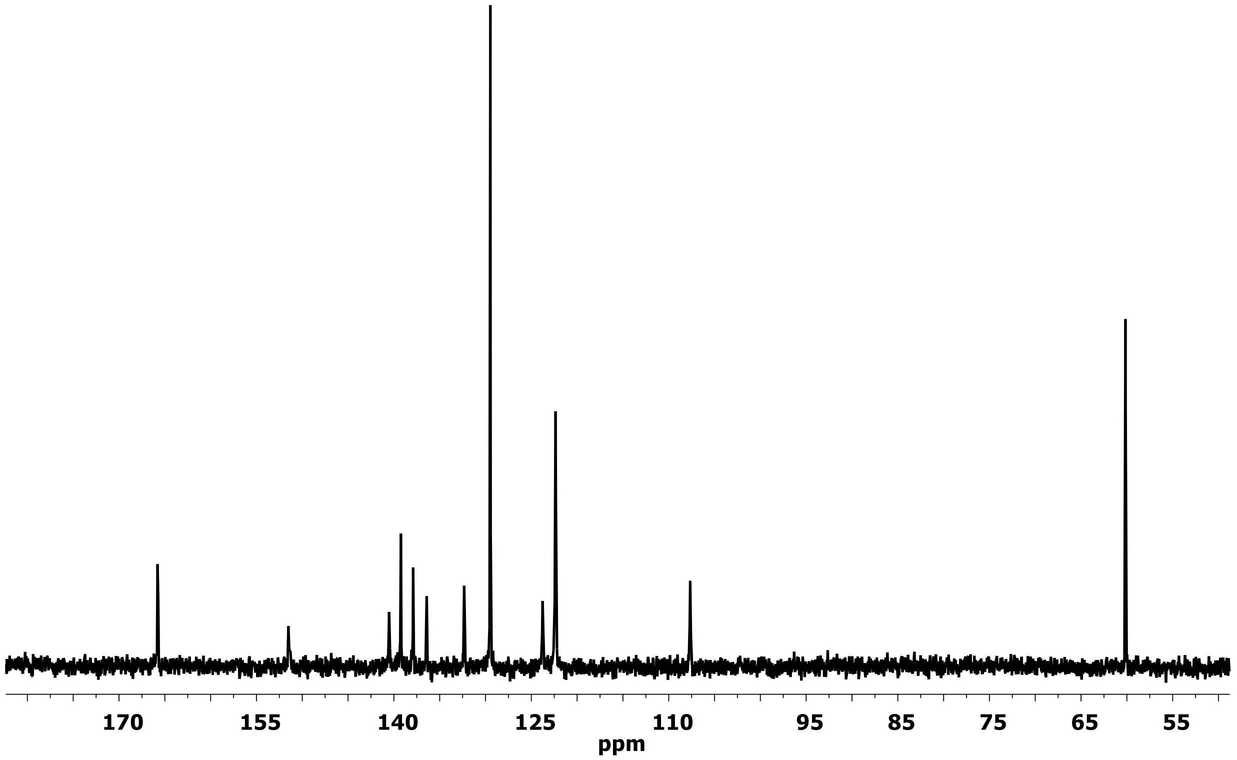

Figure S4. ^{13}C NMR spectrum of the compound **PTZa**, DMSO-d_6, 300 K, 100 MHz.

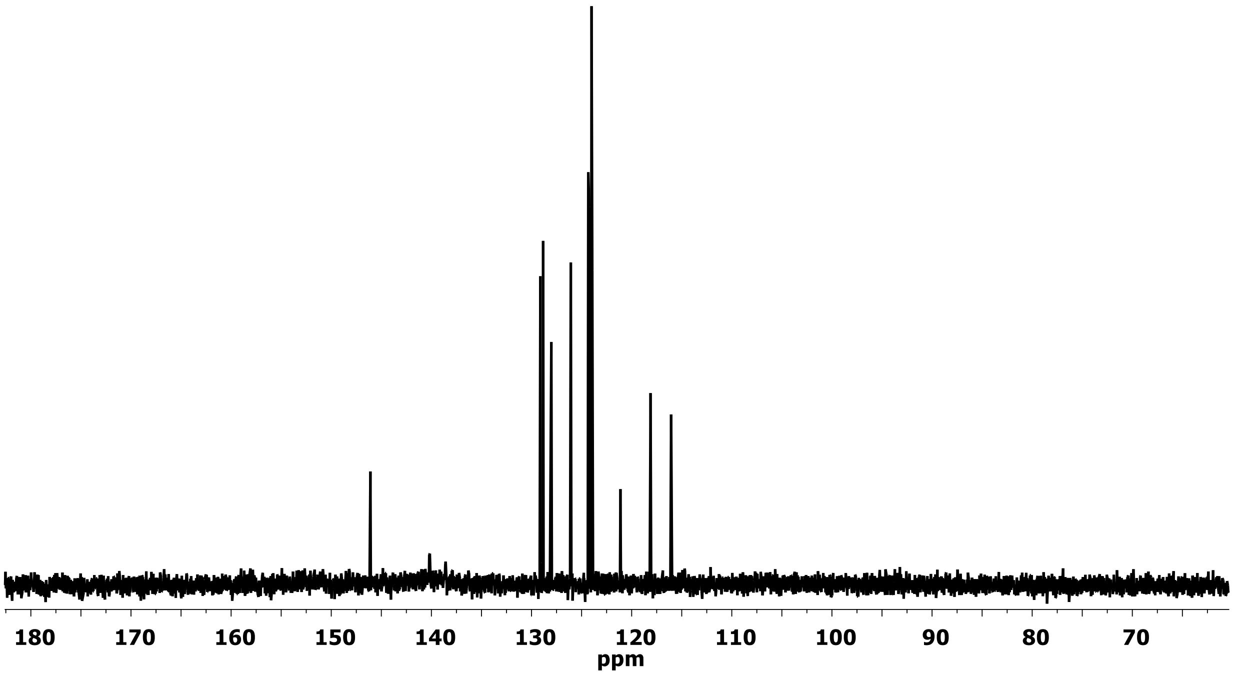

Figure S5. ^{13}C NMR spectrum of the compound **PTZ1**, DMSO-d_6, 300 K, 100 MHz.

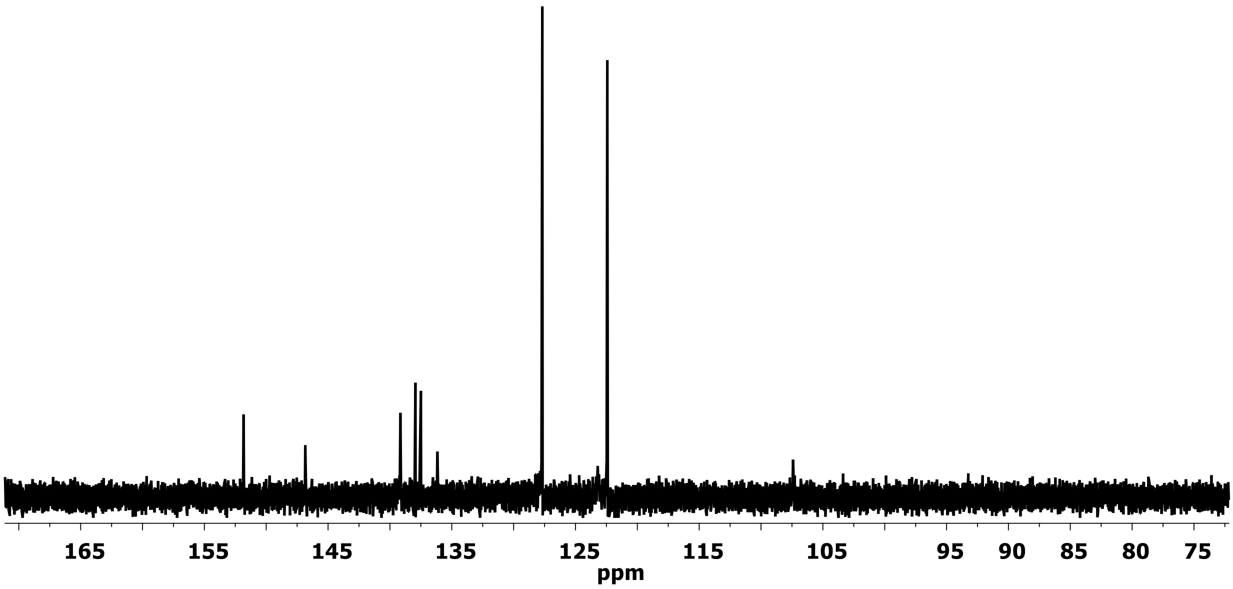

Figure S6. ^{13}C NMR spectrum of the compound **PTZ2**, DMSO-d_6, 300 K, 100 MHz.

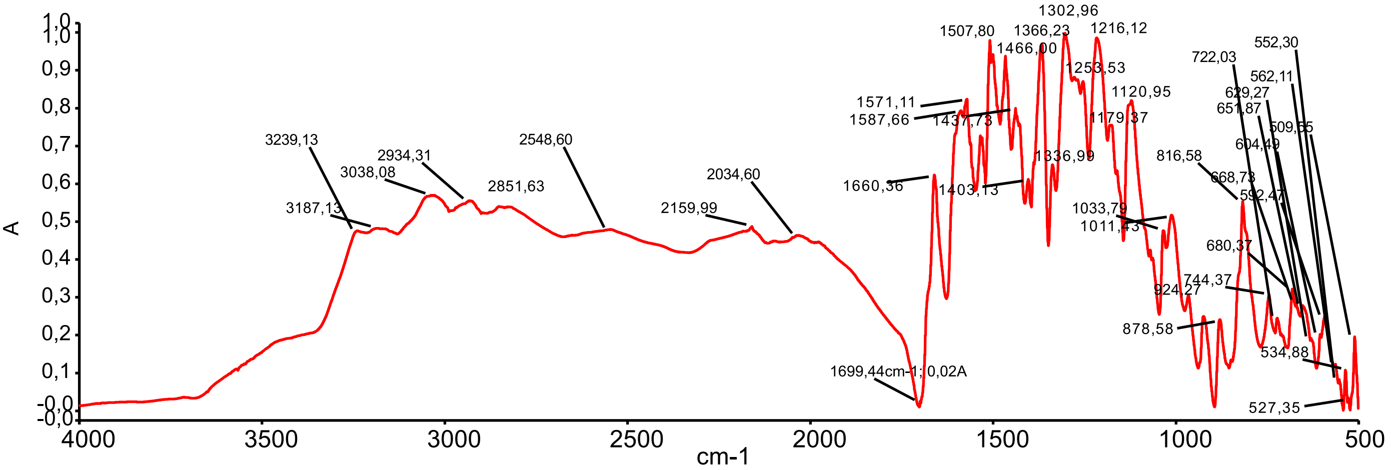

Figure S7. FT-IR spectrum of the compound **PTZa**.

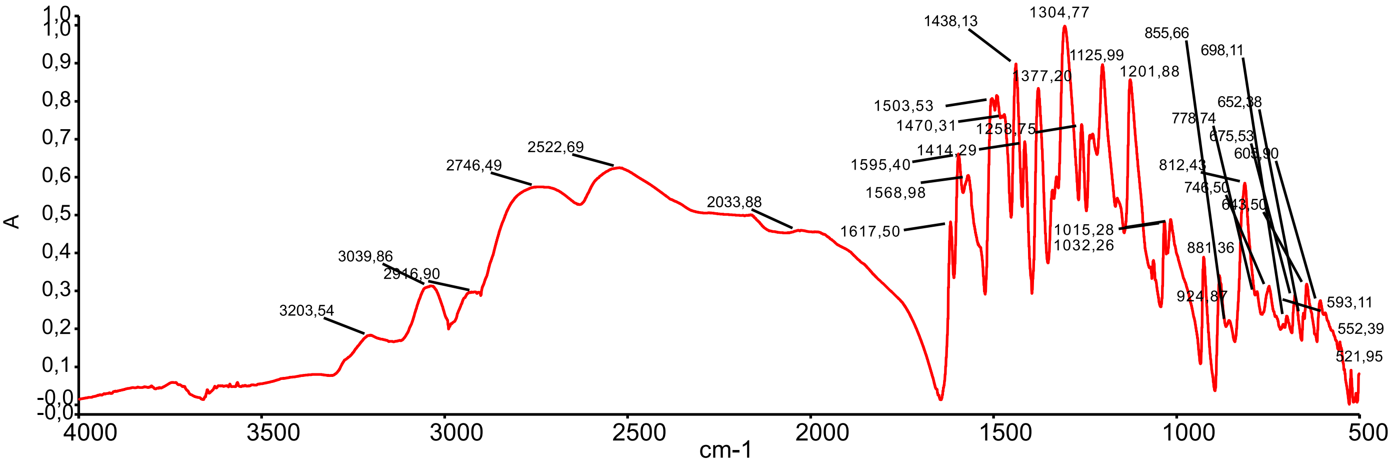

Figure S8. FT-IR spectrum of the compound **PTZ1**.

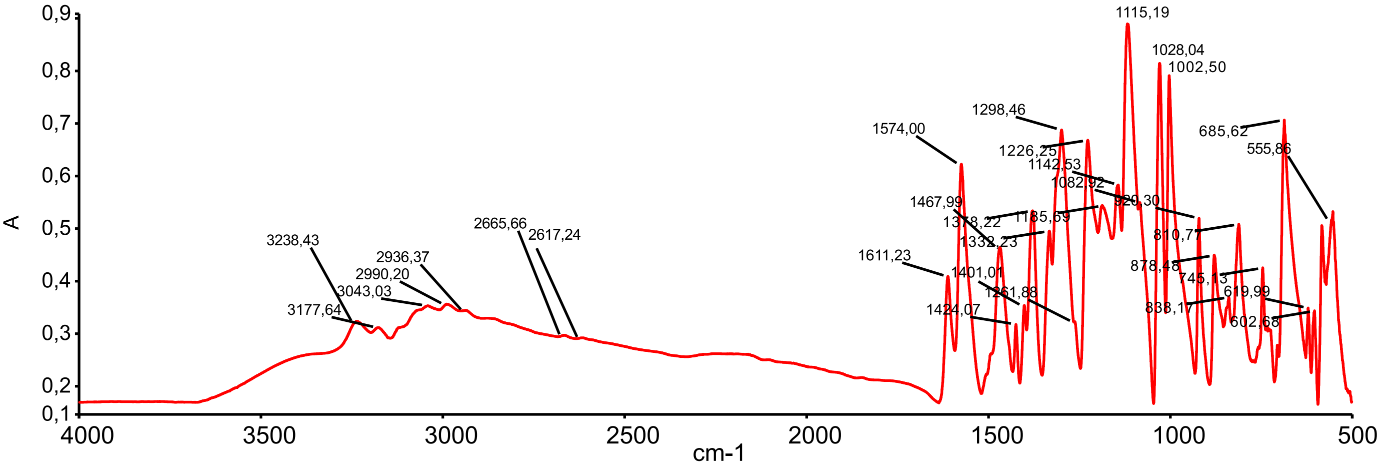

Figure S9. FT-IR spectrum of the compound **PTZ2**.

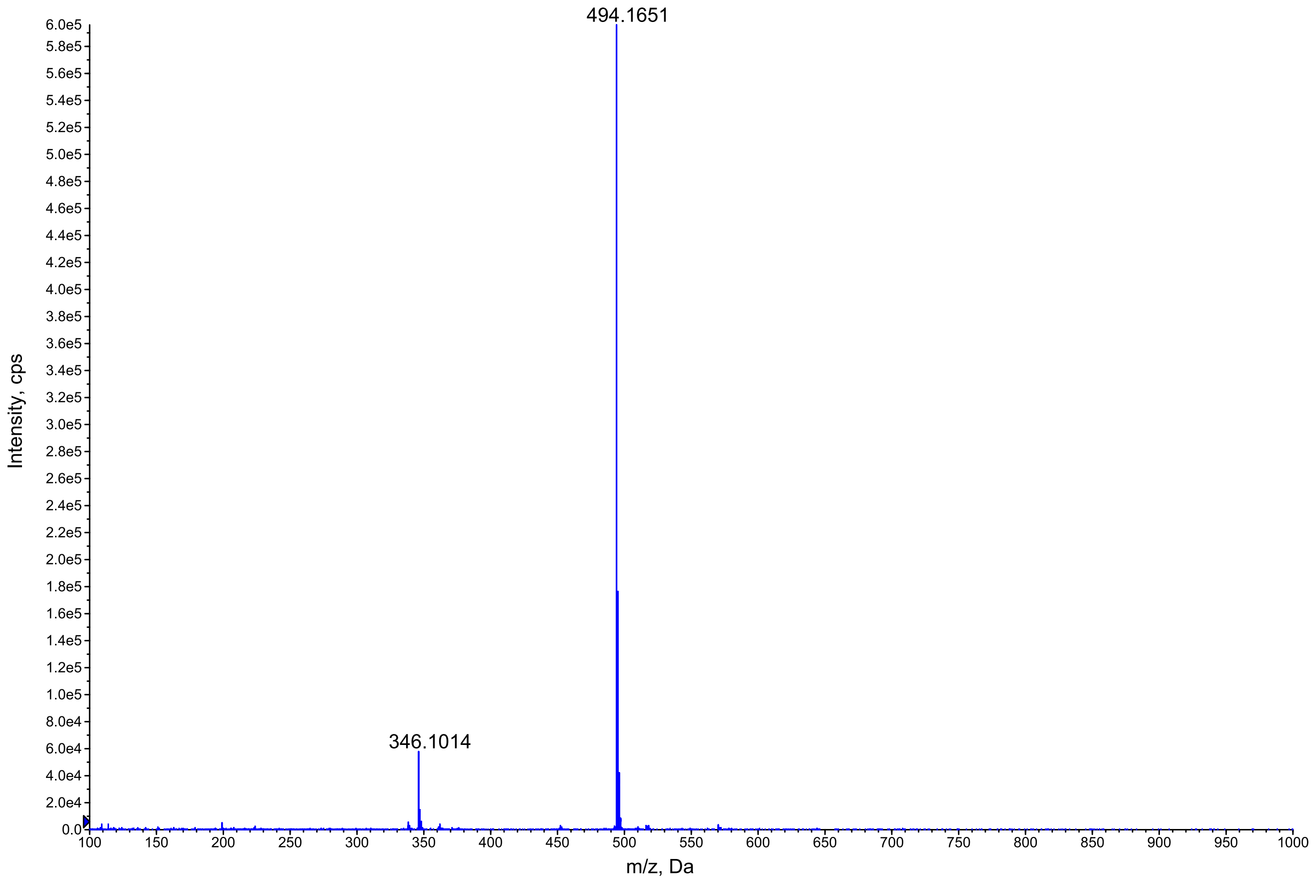

Figure S10. HRMS spectrum of the compound **PTZa**.

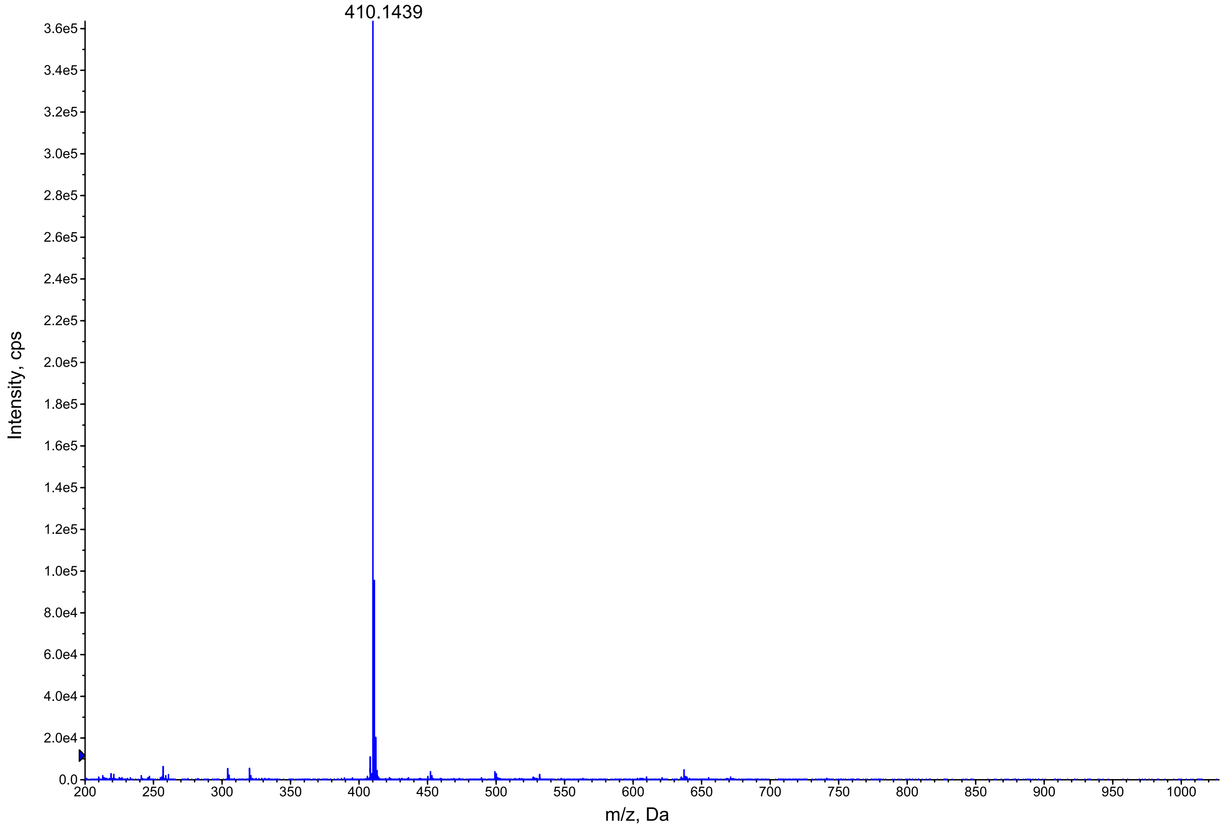

Figure S11. HRMS spectrum of the compound **PTZ1**.

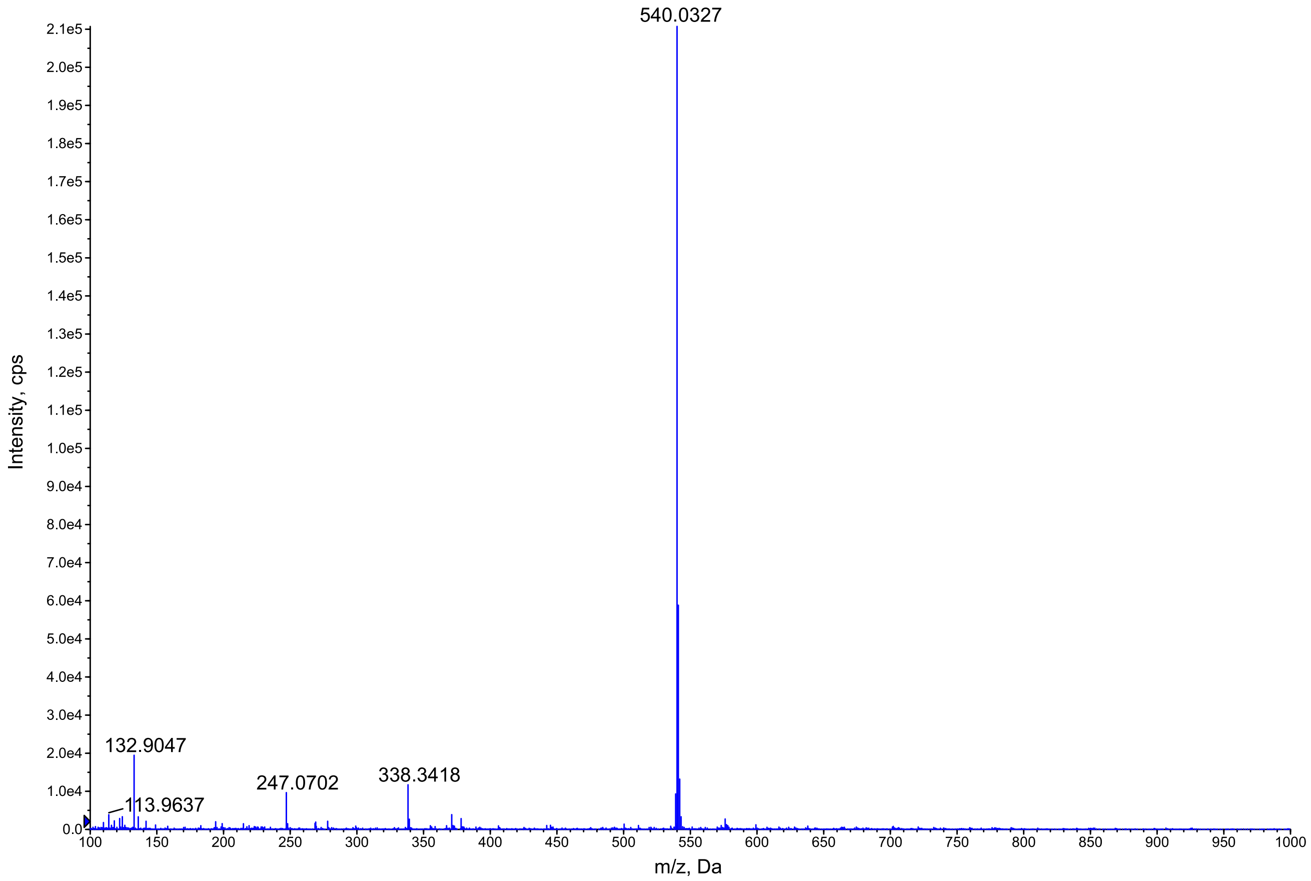

Figure S12. HRMS spectrum of the compound **PTZ2**.

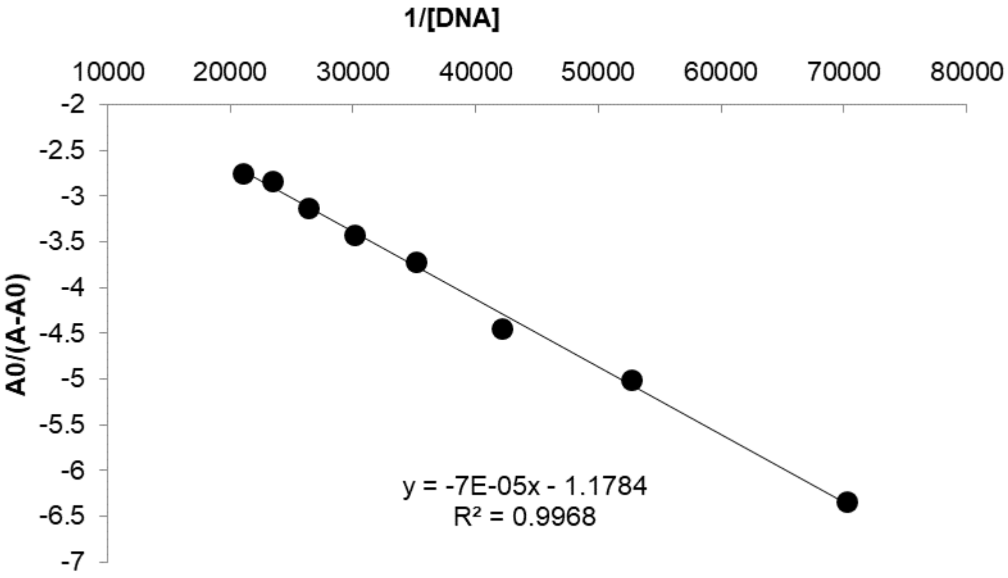

Figure S13. 1/[DNA] versus $A_0/(A- A_0)$ plot for **MB**-DNA UV-Vis titration.

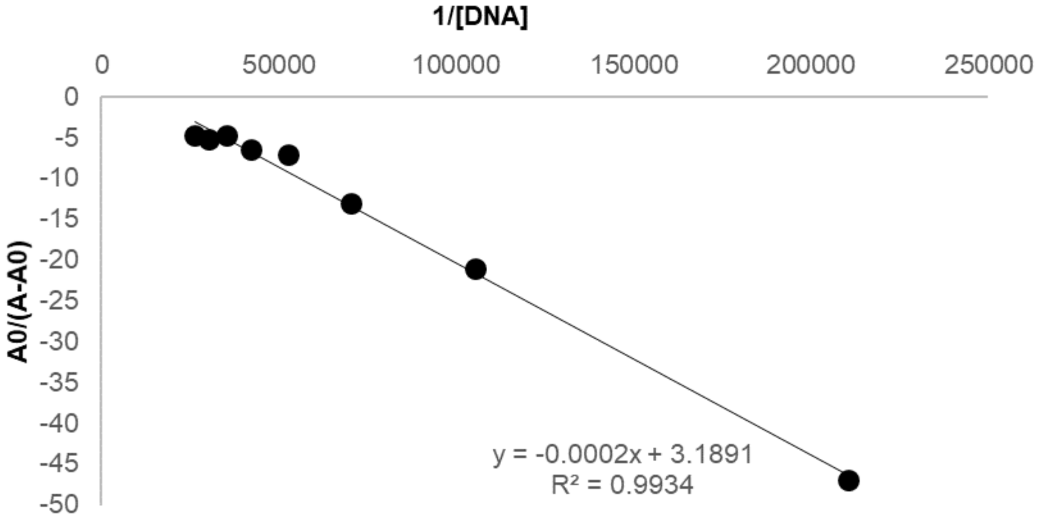

Figure S14. 1/[DNA] versus $A_0/(A- A_0)$ plot for **PTZ1**-DNA UV-Vis titration.
